# Fracture Resistance of Zirconia Restorations with a Modified Framework Design

**Published:** 2017-11

**Authors:** Sakineh Nikzadjamnani, Abbas Azari, Somayeh Niakan, Seyedeh Fatemeh Namdar

**Affiliations:** 1 Associate Professor, Dental Research Center, Dentistry Research Institute, Tehran University of Medical Sciences, Tehran, Iran; Department of Prosthodontics, School of Dentistry, Tehran University of Medical Sciences, Tehran, Iran; 2 Assistant Professor, Department of Prosthodontics, School of Dentistry, Tehran University of Medical Sciences, Tehran, Iran; 3 Assistant Professor, Dental Materials Research Center, Mashhad University of Medical Sciences, Mashhad, Iran

**Keywords:** Zirconium, Failure, Prosthesis Design, Dental Ceramic

## Abstract

**Objectives:**

Chipping is one of the concerns related to zirconia crowns. The reasons of chipping have not been completely understood. This in-vitro study aimed to assess the effect of coping design on the fracture resistance of all-ceramic single crowns with zirconia frameworks.

**Materials and Methods:**

Two types of zirconia copings were designed (n=12): (1) a standard coping (SC) with a 0.5mm uniform thickness and (2) a modified coping (MC) consisted of a lingual margin of 1mm thickness and 2mm height connected to a proximal strut of 4mm height and a 0.3mm-wide facial collar. After veneer porcelain firing, the crowns were cemented to metal dies. Afterwards, a static vertical load was applied until failure. The modes of failure were determined. Data were calculated and statistically analyzed by independent samples T-test. P<0.05 was considered statistically significant.

**Results:**

The mean and standard deviation (SD) of the final fracture resistance equaled to 3519.42±1154.96 N and 3570.01±1224.33 N in SC and MC groups, respectively; the difference was not statistically significant (P=0.9). Also, the mean and SD of the initial fracture resistance equaled to 3345.34±1190.93 N and 3471.52±1228.93 N in SC and MC groups, respectively (P=0.8). Most of the specimens in both groups showed the mixed failure mode.

**Conclusions:**

Based on the results, the modified core design may not significantly improve the fracture resistance.

## INTRODUCTION

All-ceramic crowns with zirconia frameworks have replaced the previously popular metal-ceramic crowns due to excellent aesthetics, biocompatibility, and chemical durability. In the past, glass or alumina ceramics were used in anterior restorations. However, by the development of zirconia copings, the manufacturers claim that these ceramics have a high strength [1]. It has been shown that crowns with yttria-stabilized tetragonal zirconia polycrystal (Y-TZP) cores have a half-life comparable to that of metal-ceramic crowns, and may remain in clinical service for 20 years [2]. Also, clinical studies have revealed that ceramic crowns with zirconia copings have a suitable long-term performance [3]. Despite the optimal mechanical properties, the high risk of chipping in the porcelain veneer is the main shortcoming of all-ceramic zirconia restorations [4–6]. Several reasons have been described for porcelain veneer chipping such as the zirconia sintering process, structural defects [7], damage due to grinding during the laboratory processes [8], differences in the coefficients of thermal expansion (CTE), speed of cooling, thickness of the zirconia crown [9–11], sandblasting of the zirconia crown [12, 13], framework design [3, 14], type of finish line [15], cementation process [16] and zirconia aging [17].

Among these, the framework design has not received much attention even though it may significantly affect the fracture resistance of the veneering porcelain. Although the performance of all-ceramic restorations is usually comparable to that of the porcelain-fused-to-metal (PFM) restorations, the modifications of framework design that have long been proposed for the PFMs might be helpful in improving the mechanical characteristics of all-ceramic crowns and increasing the survival rate of the restoration [18, 19]. On the other hand, the noble mechanical properties of zirconia allow practitioners to apply changes in the preparation strategies related to the coping design such as reducing the thickness from 0.5mm to 0.3mm and changing the finish line design from the chamfer to knife edge [5]. Also, a zirconia collar can be applied to support the porcelain veneer. It seems that zirconia collar extension to interproximal areas may be useful to restrict the porcelain veneer chipping and fracture in PFM crowns [6]. If the porcelain veneer has a uniform thickness and supports the lateral and compressive forces, the porcelain veneer fracture may be prevented [4].

Different types of framework designs have been suggested and their effects on the fracture resistance of all-ceramic crowns have been evaluated [3]. However, the traditional trestle design with a high lingual shoulder connected to a proximal elongated strut suggested for metal-ceramic restorations [20], has not been yet evaluated for use in all-ceramic restorations. This study aimed to evaluate the effect of a modified framework design on the fracture resistance of all-ceramic zirconia restorations in comparison with the traditional framework design. The null hypothesis was that the framework design does not influence the fracture resistance of zirconium oxide posterior single crowns.

## MATERIALS AND METHODS

**Fig. 1:**
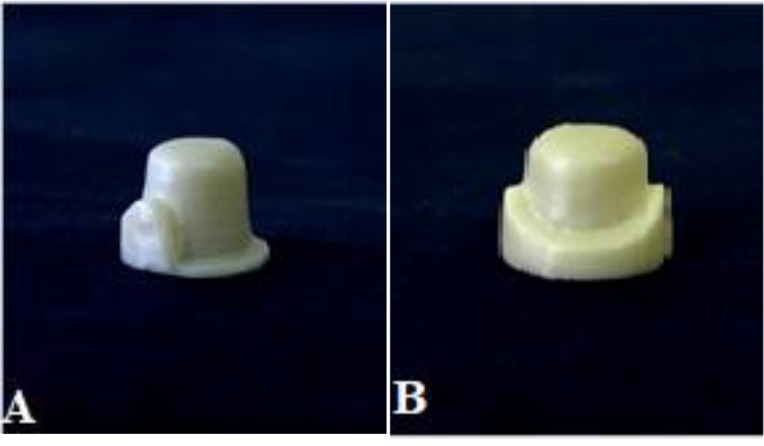
Sintered modified core (MC) design: (A) Proximal view, (B) Lingual view

In this experimental laboratory study, a standard stainless steel die with the diameter of 14mm and a total length of 40mm was prepared to achieve an occluso-gingival length of 7.5mm, a diameter of 7mm, a finish line of 1.5mm and a taper of 6° at each side. The finish line design was the radial shoulder. The prepared sample was connected to the die jig via a step, measuring 2mm in diameter and 2mm in height. An anti-rotation groove measuring 7mm in height (0.5 mm above the finish line) and 1mm in width was also prepared. The die impression was made using the silicone impression material (Speedex, Coltene/Whaledent, Switzerland) and was poured with acrylic resin (GC Pattern Resin, Tokyo, Japan). The casting of the fabricated acrylic resin die was performed using nickel-chrome (Ni-Cr) alloy (VeraBond, Aalbadent, Fairfield, CA, USA). A total of 24 dies were fabricated of Ni-Cr alloy. Table 1 shows the composition of the materials used in this study. The main die was scanned with the Cercon Eye® scanner (Cercon, DeguDent, Hanau, Germany), and a three-dimensional model of the die was fabricated; the thickness of the cement space was considered to be 30μm covering 86% of the prepared die surface (the finish line was not covered with cement and was in direct contact with the die). Twenty-four zirconia copings (Cercon, DeguDent, Hanau, Germany) were made of pre-sintered zirconia blocks with two different designs using the data obtained by scanning the die. Since the objective was to determine the effect of the coping design on the fracture resistance, in order to eliminate the effect of the interfering factors such as the connector design, zirconia crowns were used instead of zirconia bridges. The zirconia copings were divided into two groups based on their designs: a Standard coping (SC) design (n=12) with a 0.5mm uniform thickness, and a Modified coping (MC) design (n=12) with a facial collar (0.3mm in thickness and 0.3mm in height) and a buttressing shoulder of 1mm thickness and 2mm height at the lingual surface, which was increased to 4mm of height in the proximal half to form a proximal strut. Other areas of the coping were 0.5mm thick (Fig. 1). The final sintering of the pre-sintered zirconia copings was carried out in the Cercon sintering machine (DeguDent, Hanau, Germany) at 1450°C for 5 hours. Upon completion, the internal surfaces of the copings were cleaned with a cotton pellet soaked in alcohol and also with a steam cleaner. The adaptation of the coping with the die was examined using a light body silicone paste (Fit Checker, GC America Inc., Alsip, IL, USA). The interfering points on the die were relieved using a round diamond bur (D+Z, Frankfurt, Germany). The A2 shade of the Cercon Ceram Kiss porcelain (DeguDent, Hanau, Germany) was applied in a 1mm uniform thickness and was fired in two steps. An index of the first porcelain-veneered coping was made using a transparent template, which was used for the fabrication of other crowns.

**Table 1.** Composition of the materials used in the present study

**Material**	**Composition**	**Manufacturer**
Panavia F2.0 cement	Paste A: MDP, hydrophobic aromatic dimethacrylates, hydrophobic aliphatic dimethacrylates, hydrophilic aliphatic dimethacrylates, silanated silica filler, silanated colloidal silica, dl-camphorquinone, initiators Paste B: hydrophobic aromatic dimethacrylates, hydrophobic aliphatic dimethacrylates, hydrophilic aliphatic dimethacrylates, silanated barium glass filler, initiators, accelerators, pigments	Kuraray Dental, Tokyo, Japan
Nickel-Chrome casting alloy	Ni 77.95%, Cr 12.60%, Mo 5%, Al 2.9%, Co 0.45%, Be 1.95%	Verabond, Aalbadent, USA
Zirconia coping	ZrO_2_, Y_2_O_3_, HFO_2_, SiO_2_, Al_2_O_3_	Cercon, Degudent, Hanau, Germany
Ceram Kiss porcelain	SiO_2_, Al_2_O_3_, K_2_O, Na_2_O, and silicate glasses	Cercon, Degudent, Hanau, Germany
Fit checker Advanced Blue	Vinyl Polyether Silicone (VPES)	GC America Inc., Alsip, IL, USA
Self-curing acryl	Polymethyl Methacrylate (PMMA)	Acropars, Marlic Medical Co., Tehran, Iran
Pattern resin LS 1:1 Package Pattern resin LS Liquid Refill	Self-curing acrylic die material	GC America Inc., Alsip, IL, USA
Heavy body silicone putty	C-silicone	Speedex, Coltene/Whaledent AG, Switzerland

After ensuring the complete seating of the copings, the dies and crowns were separately cleaned in an ultrasonic bath and were dried. In order to remove all debris, the internal surfaces of the crowns were cleaned using 37% phosphoric acid. The Panavia F2.0 cement (Kuraray Dental, Tokyo, Japan) was prepared according to the manufacturer’s instructions and was applied to the crowns. The crowns were then seated on the corresponding dies with a mild finger pressure, and a constant pressure of 15N was applied on the specimens using a 0.5kg weight via an acrylic connector fabricated equal to the size of the crowns. After 2 to 3 seconds of light-curing and removal of excess cement, the crown margins were protected using an air-inhibiting material (OxyGuard, Kuraray Dental, Tokyo, Japan) until the cement was completely set (3 minutes). The specimens were then subjected to 5000 thermal cycles (Vafaei Industrial, Iran) between 5–55°C (20 seconds in a hot bath, 10 seconds of dwell time, and 20 seconds in a cold bath). Afterwards, the specimens were mounted in a self-polymerizing acrylic resin.

The point of load application, at the center of the occlusal surface, was marked on the specimens which were placed in the universal testing machine (Zwick/Roell, Germany). The load was applied at a crosshead speed of 1mm/minute with a round tip of 4mm diameter.

The fracture load was recorded at two phases: the initial fracture and final fracture. When the first fracture occurred, it was recorded as the “initial fracture” [1, 3], and loading was continued until the catastrophic fracture occurred [5, 19, 21, 22]; thus, the “final fracture” was measured. The mean fracture load was calculated. Statistical analysis was performed using independent samples T-test.

To ensure the accuracy, the specimens were evaluated under a stereomicroscope (Nikon, Tokyo, Japan) after the final fracture to determinate the mode of failure (at the cement-core and core-veneer interfaces). The modes of failure were divided into three categories: Adhesive failure (failure at the veneer-core or core-cement interfaces), cohesive failure (failure within the cement layer, core layer or veneer layer) and mixed failure (a combination of both adhesive and cohesive failures in different areas).

## RESULTS

**Fig. 2:**
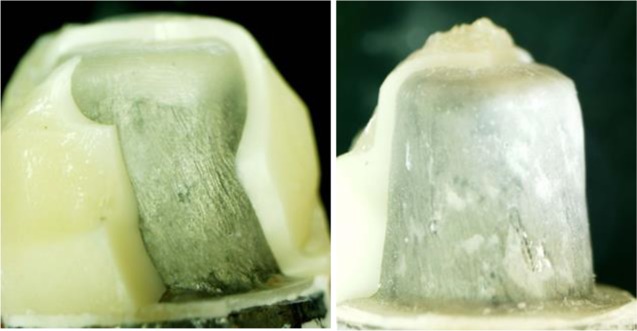
External surface of a fractured specimen with a standard core (SC) design at ×10 magnification

**Fig. 3:**
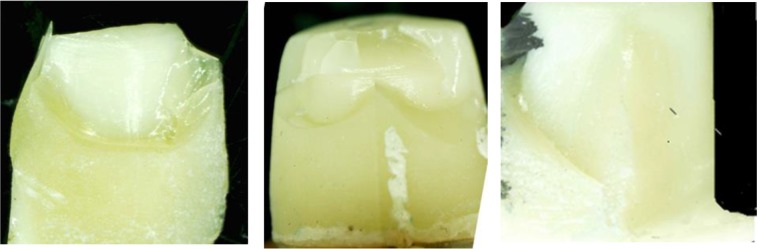
External surface of a fractured specimen with a modified core (MC) design at ×10 magnification

The means and standard deviations (SD) of the initial and final fracture resistance in the MC design and SC design are shown in Table 2. Independent samples T-test revealed no statistically significant differences between the two coping designs, neither in the initial (P=0.8) nor in the final (P=0.9) fracture resistance. Table 3 shows the failure modes of the specimens at ×63 magnification. Figures 2 and 3 show the specimens under the stereomicroscope.

**Table 2.** Mean ± standard deviation (SD) of the load (N) at fracture (the fracture resistance)

**Fracture resistance**	**Modified coping**	**Standard coping**	**P-value**
Initial	3471.52±1228.93	3345.34±1190.93	0.8
Final	3570.01±1224.33	3519.42±1154.96	0.9

**Table 3.** Frequency and percentage of the failure modes at ×63 magnification

		**Mixed N(%)**	**Cohesive N(%)**	**Adhesive N(%)**
**Modified coping**	Die-Core	11(92)	0	1(8)
	Core-Veneer	5(42)	1(8)	6(50)
**Standard coping**	Die-Core	9 (75)	0	3(25)
	Core-Veneer	7(59)	3(25)	2(16)

## DISCUSSION

The changes applied to the core design of the present study were based on the trestle design suggested for metal-ceramic crowns and were aimed to decrease the risk of fracture in PFM restorations. Miller [18] stated that the trestle design of the metal copings for single crowns must include a proximal strut, a buttressing shoulder, a reinforcing collar, shear resistant cusps, and abutment seal. Since the trestle design is believed to be optimal for metal-ceramic crowns, in the current study, this design was used for zirconia copings to assess its efficacy for all-ceramic zirconia restorations.

The shear strength is achieved by creating an anatomical form on the occlusal surface of copings. This topic has been evaluated in previous studies and its positive effect on the fracture resistance of restorations has been well-confirmed [1, 3, 21, 22]. In the current study, the fracture resistance of the MC group was higher than that of the SC samples, but this difference was not statistically significant (P=0.9 in the final fracture and P=0.8 in the initial fracture). Moreover, the load at the initial fracture in the SC group was lower than that at the initial fracture in the MC group. Veneer fracture occurred more frequently in the SC specimens (n=7) under lower levels of stress. However, in the MC group, ceramic veneer chipping occurred in only two specimens prior to core fracture. This result may be explained by the greater veneer support provided by the MC design. Finite element analyses have also shown that compressive stresses (instead of tensile stresses) are present at the veneer-shoulder interface; thus, the support provided by the shoulder finish line could reduce the risk of veneer fracture [23, 24]. Moreover, the buttressing shoulder with an increased height serves as a shock-absorber at the areas near the crown margins; this area provides a great support for the veneering porcelain under functional loads [25].

In 2004, Sundh and Sjogren [22] concluded that the adapted core framework design caused a greater improvement in the fracture resistance compared to the 0.5mm-thick standard design. In 2011, Kokubo et al [3] reported similar results and showed that the uniform-thickness coping required the minimum amount of fracture load. Also, the cuspal configuration of the uniform-thickness coping design and the modified coping margins increased the fracture resistance of the veneering porcelain [3]. The modified design of the coping margins adopted by Kokubo et al [3] was based on a design suggested by Marchack et al [26] in 2008. The authors recommended further modifications of the margin design (i.e. increasing the height of the collar); however, some studies reported that the modified framework design did not improve the fatigue resistance of the crowns [3, 27]. Such controversy in the results may be attributed to the differences in the type of the materials, tests, and methods of load application. Bonfante et al [19] and Silva et al [28] also assessed the efficacy of an MC design and showed that the modified margin design provided a greater support for the porcelain. The details of the coping design, load application status, and type of the materials used in the two above-mentioned studies were different from those of our study. The mode of failure in both groups of the current study was mainly the mixed fracture. Adhesive failure (at the core-veneer interface) was more frequent in the MC group (n=6) compared to the SC samples (n=2). In all the specimens, the fracture initiated at the site of load application and extended laterally. In the MC group, the fracture line extended toward the borders of the proximal strut and did not involve the buttressing shoulder or the proximal strut body. Conversely, in the SC group, the fracture extended toward the margins; these findings were in accordance with those of Bonfante et al [29].

It seems that proximal struts provide greater support for the veneer and prevent the crack propagation. Clinical fractography shows that the crown’s margins are susceptible to crack formation since high amounts of stress are concentrated at the margins [6, 27]. Thus, the SC design with a uniform thickness may serve as a weak point in all-ceramic restorations. On the other hand, the differences in the modes of failure among various studies may be attributed to the different types of ceramics, die materials, manufacturing techniques, specimen designs, and various thicknesses of the walls and luting agents [30]. It is suggested to predefine the location of failure assessment in subsequent studies to standardize the observation area under the stereomicroscope.

Similar to previous studies [3, 31], the static load was used to assess the fracture resistance in this study. Although the fracture strength test is highly important for ceramic restorations, static load application has some limitations and does not perfectly simulate the clinical condition. It does not provide any information on the long-term behavior of the materials or their properties when exposed to cyclic fatigue in the oral environment. Moreover, the complex oral environment and the role of factors such as saliva and patient-related habits, etc. cannot be ideally simulated by the current laboratory techniques. These items are among the limitations of this study. Therefore, the results of static tests must be cautiously interpreted [30, 32].

In the current study, similar to that of Beuer et al [20], standard metallic dies were used for the fabrication of specimens. According to Schererr and de Rijk [30], the higher elastic modulus of the die resulted in a higher fracture resistance. The elastic modulus of metallic dies is much higher than that of dentin (200 GPa versus 18.3 GPa) [20]; therefore, these dies undergo a limited transformation and as a result, low shear stresses are created in the internal surfaces of the crowns; this is considered an advantage [30]. Thus, the fracture resistance values obtained by the application of these dies may be much higher than those of the dentinal dies. On the other hand, metallic dies have a significant role in standardizing the preparations and obtaining crowns with identical physical qualities. These are among the positive points of the current study. All the crowns in our study were cemented to metallic dies using the Panavia F2.0 resin cement. It has been stated that the fracture resistance of the all-ceramic crowns cemented with resin-based cements is higher than that of the crowns luted by conventional cements [33].

## CONCLUSION

There were no statistically significant differences in the fracture resistance between the modified and standard coping designs, neither in the initial nor in the final fracture strength. Most of the specimens in both groups showed the mixed failure mode. Based on the results, the modified core design may not significantly improve the fracture resistance of zirconia restorations.
